# Effect of trimetazidine against ovarian ischemia/reperfusion injury in rat model: A new pathway: JAK2/STAT3

**DOI:** 10.22038/IJBMS.2023.72544.15776

**Published:** 2023

**Authors:** Tugba Nurcan Yuksel, Zekai Halici, Elif Cadirci, Erdem Toktay, Bengül Ozdemir, Ayşe Bozkurt

**Affiliations:** 1 Department of Pharmacology, Faculty of Medicine, Tekirdag Namık Kemal University, Tekirdag, Türki̇ye; 2 Department of Pharmacology, Faculty of Medicine, Ataturk University, Erzurum, Türki̇ye; 3 Clinical Research, Development and Design Application and Research Center, Ataturk University, Erzurum, Türki̇ye; 4 Department of Histology and Embryology, Faculty of Medicine, Kafkas University, Kars, Türki̇ye; 5 Department of Pharmacology, Faculty of Pharmacy, Van Yuzuncu Yıl University, Van, Türki̇ye

**Keywords:** Ischemia, JAK2/STAT3, Oxidative stress, Ovary, Reperfusion, Trimetazidine

## Abstract

**Objective(s):**

Ovarian ischemia/reperfusion (I/R) is an extremely complex pathological problem that begins with oxygen deprivation, progresses to excessive free radical production, and intensifies inflammation. The JAK2/STAT3 signaling pathway is a multipurpose signaling transcript channel that plays a role in several biological functions. Trimetazidine (TMZ) is a cellular anti-ischemic agent. This study aims to investigate the effects of TMZ on ovarian I/R injury in rats.

**Materials and Methods:**

sixty four rats were divided into 8 groups at random: healthy(group1); healthy+TMZ20(group2); ischemia (I) (group 3); I+TMZ10(group4); I+ TMZ20(group5); I/R(group6); I/R+TMZ10(group7); I/R+TMZ20(group8). Vascular clamps were placed just beneath the ovaries and over the uterine horns for 3 hr to induce ischemia. The clamps were removed for the reperfusion groups, and the rats were reperfused with care to ensure that the blood flowed into the ovaries, subjecting them to reperfusion for 3 hr. TMZ was administered orally by gavage 6 and 1 hr before operations. At the end of the experiment, ovarian tissues were removed for biochemical, molecular, and histopathological investigation.

**Results:**

TMZ administration ameliorated ischemia/reperfusion-induced disturbances in GSH and MDA levels. TMZ treatment inhibited I/R-induced JAK2/STAT3 signaling pathway activation in ovarian tissues. TMZ administration also improved the increase in the mRNA expressions of IL-1β, TNF-α, and NF-κB caused by ischemia/reperfusion injury. Moreover, TMZ treatment improved histopathologic injury in ovarian tissues caused by ischemia/reperfusion.

**Conclusion:**

TMZ treatment protected rats against ovarian ischemia/reperfusion injury by alleviating oxidative stress and inflammatory cascades. These findings may provide a mechanistic basis for using TMZ to treat ovarian ischemia-reperfusion injury.

## Introduction

Ovarian torsion is defined as the twisting of the ovary around its promoting ligaments, which prevents blood flow to the ovary, leading to ischemia, necrosis, and irreparable damage (1). However, reperfusion following a period of ischemia creates a new pathophysiological process that can lead to more tissue damage. This process is named ischemia-reperfusion (I/R) injury (2). Reperfusion following detorsion raises free radicals, cytokines, nitric oxide, neutrophil activation, and apoptosis. As a consequence, oxidative damage caused by reperfusion, known as I/R injury, develops in the ovaries, which may be more damaging than ischemic injury (3).

Oxidative stress is a major contributor to ovarian I/R-induced damage. I/R generates free radicals and reactive oxygen species (ROS), which cause oxidative stress, inflammation, and apoptosis (4, 5). Because of their electronically unstable and ionized atomic structure, ROS, which are derived from molecular oxygen, can appear as free radicals or in other forms. These molecules interact with biological macromolecules by enticing electrons and interfering with their biological functionality (6, 7). The production of ROS involves a number of enzymatic processes, including the reduction of dioxygen (O_2_) in the mitochondria, which can result in several intermediate forms of ROS; the reduction of amino acid oxidase in peroxisomes, which causes oxidative deamination of -keto acids; and the production of ROS by copper and iron ions, which can then be released into the bloodstream by ceruloplasmin, transferrin, and albümin (7). Also, the generated ROS triggers lipid peroxidation (with malondialdehyde (MDA), generated as the end product) (8). Increased MDA levels with lipid oxidation damage cell membrane functions and cellular integrity (9, 10). Increasing the concentrations of nonenzymatic compounds such as glutathione (GSH) stimulates the cellular defense system against oxidative damage (11).

The JAK-STAT signaling pathway is a multipurpose signaling transcript channel that plays a role in several biological functions, such as immune control, cell differentiation, proliferation, and apoptosis (12, 13). Particularly in the I/R paradigm, activation of the JAK/STAT signaling pathway hub increases cellular apoptosis, and inflammatory and oxidative stress responses (14). Consequently, pharmacological agents with multiple effects such as anti-oxidative, anti-inflammatory, anti-apoptotic (15), and anti-necrotic (16) properties may be a promising strategy for avoiding I/R-induced ovarian tissue injury.

Trimetazidine [1-(2,3,4-trimethoxybenzyl) piperazine dihydrochloride] (TMZ) is a piperazine-derived agent (17). TMZ is a cellular anti-ischemic agent that blocks the mitochondrial long-chain 3-ketoacyl coenzyme a thiolase enzyme. Also, TMZ promotes the metabolism of mitochondria by blocking myocardial fatty acid uptake and oxidation, which stimulates glucose oxidation (18). Compared with traditional anti-ischemic drugs, trimetazidine exerts direct effects on myocardial ischemia without inducing hemodynamic changes (19). Previous studies have shown TMZ has beneficial effects in animal models of oxidative stress (20), apoptosis (21), and inflammation (22)-related disorders. Considering these pharmacological characteristics of TMZ, in different experimental studies, the protective effects of TMZ have been individually proven in various tissues such as renal (23), hind-limb (24), cerebral (25), and myocardial (26) I/R injury. Moreover, there are limited studies investigating the impact of TMZ on ovarian I/R injury in the literature (27, 28). As far as we know, the present study is the first and most comprehensive study to investigate the effects of TMZ on ovarian I/R injury which differs from those in the literature with its features; a) in separate groups in rats with both ischemia and I/R injury, b) effects of two different doses of TMZ, c) determining mRNA expression levels of tumor necrosis factor-alpha (TNF-α), and d) impacts of TMZ on JAK2/STAT3 signaling pathway, an important mediator in I/R injury, with quantitative real-time polymerase chain reaction (qRT-PCR) analyses. 

Based on all this information, in this study, we aim to investigate the protective effects of TMZ on ovarian I/R injury in rats with biochemical (glutathione (GSH), and malondialdehyde (MDA) levels), molecular (qRT-PCR analyses of interleukin 1 beta (IL-1β), TNF-α, nuclear factor kappa B-p65 (NFκB-p65) and JAK2/STAT3 signaling pathway), and histopathological (staining with Harris Hematoxylin and Eosin Y) analyses.

## Materials and Methods

***Animals***

In this study, 64 female Wistar rats aged 4 –5 months (weight: 250–290 gr), were purchased from Ataturk University Medical and Experimental Application Center Experimental Animal Laboratory. All the animals were kept in standard plastic cages under standard conditions (temperature: 22 ± 1 °C, relative humidity: 40–80%, 12 hr light-dark cycle). Throughout the experiment, the animals had unlimited access to the usual rat water and food (*ad libitum*). All experimental procedures were carried out in accordance with national guidelines for the use and care of laboratory animals.

***Ethics statement***

This study and all its protocols were approved by Atatürk University Animal Experiments Local Ethics Committee (05.04.2022, document number E-42190979-050.01.04-2200109460).

***Chemicals***

TMZ (Vastarel MR 80 mg, 30 capsules) was purchased from Abdi İbrahim, TÜRKİYE. Xylazine (Basilazin 2%) was obtained from BioTek, TÜRKİYE. Ketamine (Ketalar 500 mg/10 mL) was obtained from Pfizer, TÜRKİYE. The lab experiments required additional chemicals, all of which were bought from Sigma and Merck (Germany).

***Experimental strategy***

**The 64 rats were divided into 8 groups at random (n** **=** **8). TMZ, in capsule form, was powdered and dissolved in distilled water. The dose **of TMZ (10 and 20 mg/kg) was determined by earlier research (27-29). TMZ was administrated by oral gavage 6 and 1 hr before the operation as the elimination half-life of the TMZ is about 6 hr, related groups are in (30, 31) (Table 1).

***Surgical procedure for inducing ischemia-reperfusion***

**A**ll animals were anesthetized with an injection of 80 mg/kg ketamine + 8 mg/kg xylazine. After disinfecting the abdominal area, a 2.5 cm longitudinal incision was made in the lower abdomen’s midline. A small peritoneal cut was performed to locate the uterine horns and adnexa. The uterine horn and ovaries were specified. Vascular clamps were placed just beneath the ovaries and over the uterine horns for 3 hr to induce ischemia. At the end of 3 hr, the ischemia groups were terminated. The clamps were removed for the reperfusion groups. Rats were reperfused for 3 hr to allow blood to flow to the ovaries. At the end of 3 hr, the reperfusion groups were terminated. The rats in the ischemia groups were euthanized with a high dose of anesthesia at the end of the 3 hr ischemia operation. The rats in the reperfusion group were euthanized with a high dose of anesthesia at the end of a total of 6 hr. All ovarian tissues were collected and kept at −80 °C to investigate biochemically and molecularly and at 10% formalin solution to investigate histopathologically.

***Biochemical investigations***

100 mg of all specimens reserved for biochemical investigations were treated with 1 ml of PBS, ground in liquid nitrogen with a Tissue Lyser II (Qiagen) and centrifuged. Supernatants obtained by centrifugation were used as samples. GSH (32) and MDA (33) levels were determined with an enzyme-linked immunosorbent assay (ELISA) reader (34). The levels of GSH and MDA in ovarian tissues were measured as nmol/mg protein. The mean and standard deviation for each set of data was displayed per mg of protein.

***Protein determination***

Utilizing commercial protein standards (Sigma Aldrich, Total protein kit-TP0300-1KT-(USA)), the Lowry technique was employed to calculate the protein concentrations (35).

***Molecular investigations***

*Gene expressions analyses*

A qRT-PCR was designed to assess IL-1β, TNF-α, NFκB-p65, JAK2, and STAT3 mRNA expression levels. To do this, ovarian tissues were homogenized, RNA was isolated, cDNA was created, and the expression levels of various mRNAs were quantitatively assessed.

*RNA extraction from ovarian tissues*

Ovarian tissue specimens were measured separately at 20 mg. Specimens were stabilized in RNAlater RNA Stabilization Reagent (Qiagen) and homogenized with Tissue LyserII (Qiagen). Using the RNeasy Mini Kit Qiagen and following the manufacturer’s instructions in Qiaqube (Qiagen, Hilden, Germany), total RNA was purified. The total amount of mRNA was determined utilizing nanodrop spectrophotometry (All Sheng) at 260 nm (36). 

*Reversed transcriptase reaction and cDNA synthesis*

cDNA production from total RNA was performed with a High Capacity cDNA Reverse Transcription Kit (Applied Biosystems, Foster City, CA, USA). 10 μl RNA was used for each reaction. cDNA synthesis was achieved with T100 Thermal Cycler (BIO-RAD) according to temperature measurements. By using nanodrop spectrophotometry (All Sheng), the quantity of cDNA was determined, and the obtained cDNA was kept at -20 °C. For the cDNA synthesis reaction, the following ingredients were used: total RNA (10 µl), 25 X dNTP mix (0.8 µl), 10X RT random primers (2 µl), reverse transcription 10X buffer (2 µl), diethylpyrocarbonate H2O (4.2 µl) and MultiScribe reverse transcriptase (1 µl). The cDNA concentrations were assessed and quantified using the Epoch Spectrophotometer System and Take3 Plate (Biotek) (37, 38).

*Quantitative Determination of IL-1β, TNF-α, NFκB-p65, JAK2, and STAT3 mRNA gene expression by Real-Time PCR*

Utilizing the StepOnePlus Real-Time PCR System technology (Applied Biosystems, USA) and cDNA produced from RNA of rats, analyses of relative and IL-1β, TNF-α, NFκB-p65, JAK2, and STAT3 expression analyses were carried out, as previously described (39). TaqMan Gene Expression Assays: Rat IL-1β (Rn00580432_m1), rat TNF-α (Rn00562055_m1), rat NFKβ (Rn01399583_m1), rat JAK2 (Rn00676341_m1) and rat STAT3 (Rn00562562_m1) primers were used for the real-time polymerase chain reaction. β-actin (housekeeping gen) (Rn00667869_m1) expression results in each tissue were used as the reference gene. The Corbett Rotor-Gene (Thermo Fisher Scientific) equipment was used for the amplification and quantification procedures. The following TaqMan® Gene Expression Assays for 100ng cDNA were pipetted for 40 cycles with 100 ng cDNA, 1 μl Assay, and 10 μl TaqMan Master Mix followed by completion to 20 μl with RNase-free H_2_O. The number of cycles at which the amount of fluorescent signal seen in qRT-PCR experiments exceeds the lowest value is known as the cycle threshold (Ct). The results were statistically analyzed after the Ct values were automatically transformed into delta delta Ct (2-∆∆Ct) (40).

*Histopathological analysis*

Preparation of solutions, dehydration and clearing procedures of tissue samples, preparation of sections, and staining with Harris Hematoxylin and Eosin Y were carried out in line with previous studies for histopathological evaluation (41). The ovarian tissue sections collected from rats for histopathological analysis were quickly fixed in a 3.7% formaldehyde (10 % formalin) solution for 48 hr. All samples for histological tissue processing were routinely processed after fixation. To remove the fixative, the tissues were washed under running water for 30 min. The tissues were passed through 70%, 80%, and 96% alcohol concentrations at increasing degrees and kept in 96% alcohol for 1 night. After the tissue samples were kept in 100% alcohol twice for 1 hr, it was passed through xylene solution series twice for 15 min. All the tissue samples were paraffin treated in three changes for 1 hr in an oven at 60 °C, and the blocking process was performed. Each paraffin block of tissue was cut to a thickness of 5 micrometers for histopathological analysis after blocking. On the slide covered in adhesive, paraffin sections were cut. All slides were then stained using Harris Hematoxylin and Eosin Y. For each ovary tissue slide, at magnifications of 10X, 20X, and 40X, at least five areas were examined. For histopathological assessments, the number of necrotic and apoptotic cells, size of edema and hemorrhage areas as well as vascular dilation in ovarian tissues were evaluated by a light microscope. The results of the Harris Hematoxylin and Eosin Y staining were evaluated using semi-quantitative scoring and the average staining density score for injury was taken into account as none: 0, mild: 1, medium: 2, severe:3, or very severe: 4 (30). 

***Statistical analysis***

For the statistical analysis of biochemical (GSH and MDA) and molecular (IL-1β, TNF-α, NFκB-p65, JAK2, and STAT3) investigations, the data were analyzed using GraphPad Prism, version 5.0, and are shown as means ± standard deviation (SD). One-Way ANOVA and Tukey’s multiple comparison tests were used to compare the groups; *P*-values ​​less than 0.05 were considered significant. Means with the same letter in the same column do not differ significantly from each other, whereas means with different letters in the same column show significant differences between the groups.

## Results

***Impacts of TMZ on oxidant and anti-oxidant parameters in ovarian tissue***

**Figure 1:**
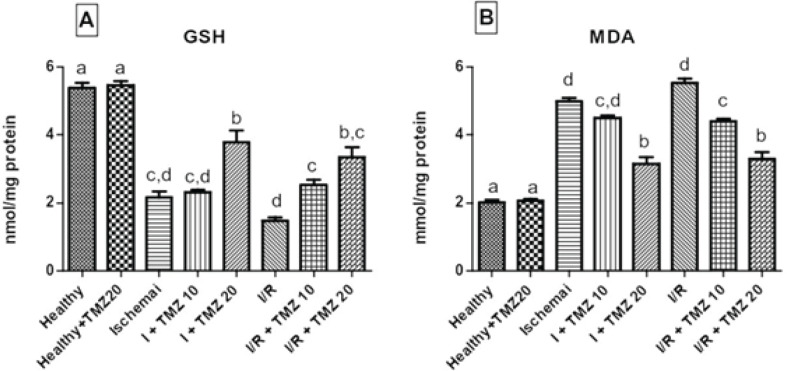
Effects of TMZ on GSH (A) levels (nmol/mg protein) and MDA (B) levels (nmol/mg protein) in ovarian I/R injury in rats

**To investigate the effect on oxidative stress parameters of TMZ, **GSH (Figure 1A) and MDA (Figure 1B) levels were analyzed. The application of 20 mg/kg TMZ only in rats having a sham operation did not affect GSH and MDA levels in comparison to the healthy group (*P*>0.05) (Figure 1A-B). When compared to the healthy group, GSH levels, which are signs of anti-oxidant capacity, significantly declined in the ischemia and I/R groups (*P*<0.05). While GSH levels of the ischemia group and I+TMZ 10 group were similar, the TMZ treatment (I+TMZ 20, I/R+TMZ 10, and I/R+TMZ 20 groups) showed a significantly fixed effect on the decrease in GSH levels caused by ischemia and I/R, in a dose-dependent manner (*P*<0.05). MDA levels, indicators of the oxidant status, rose in the ischemia and I/R groups, in comparison to the healthy group (*P*<0.05). The TMZ treatment (I+TMZ 10, I+TMZ 20, I/R+TMZ 10, and I/R+TMZ 20 groups) reduced MDA levels dose-dependently, in comparison to the ischemia and I/R groups.

***Impacts of TMZ on JAK2/STAT3 signaling pathway in ovarian I/R injury***

**Figure 2:**
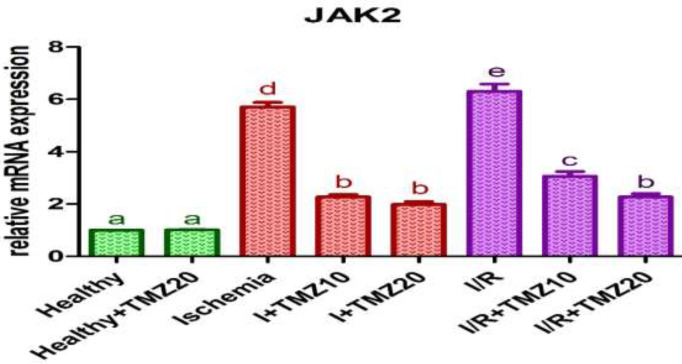
Effects of TMZ on JAK2 mRNA expression levels in ovarian I/R injury in

**Figure 3:**
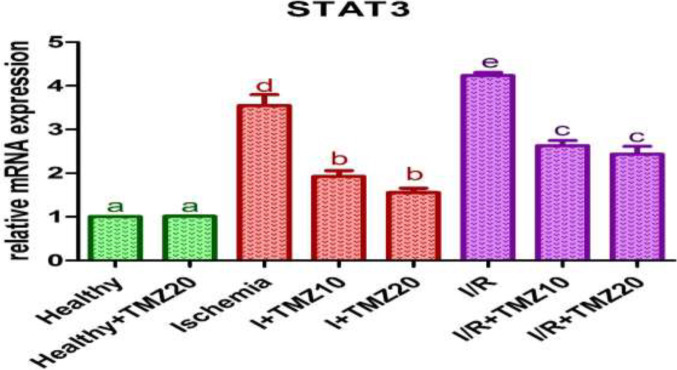
Effects of TMZ on STAT3 mRNA expression levels in ovarian I/R injury in rats

To examine the impacts of TMZ on the JAK2/STAT3 signaling pathway in ovarian I/R injury, JAK2 and STAT3 mRNA expression levels in the ovarian tissue of rats were analyzed. JAK2 (Figure 2) and STAT3 (Figure 3) mRNA expression levels in rats treated with 20 mg/kg TMZ alone and subjected to sham surgery did not differ statistically from the healthy group (*P*>0.05). JAK2 and STAT3 mRNA expression levels were significantly increased in the ischemia and I/R groups as compared with the healthy group (*P*<0.05). These levels were significantly reduced in TMZ-administered groups (I+TMZ 10, I+TMZ 20, I/R+TMZ 10, and I/R+TMZ 20), dose-dependently as compared to the ischemia and I/R groups (*P*<0.05).

***Impacts of TMZ on anti-inflammatory parameters in ovarian I/R injury***

**Figure 4:**
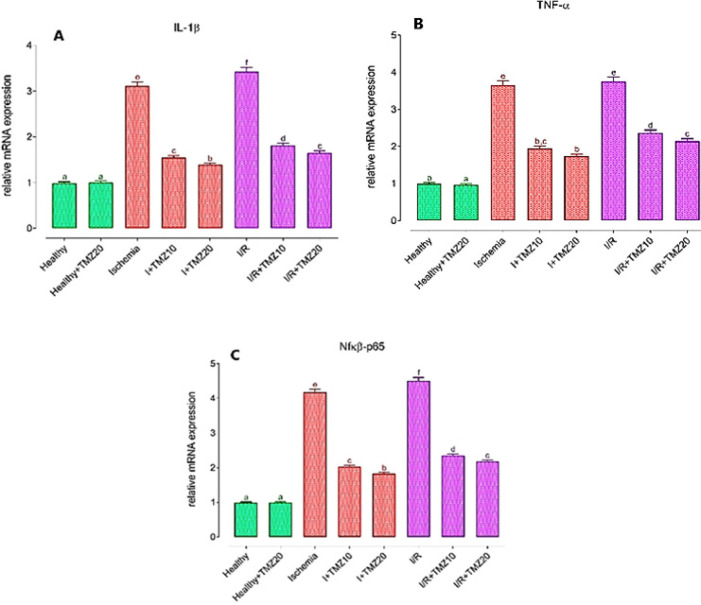
Effects of TMZ on IL-1β (A), TNF-α (B), and NFκB-p65 (C) mRNA expression levels in the ovarian I/R injury in rats

To look into the anti-inflammatory properties of TMZ, IL-1β (Figure 4A), TNF-α (Figure 4B), and NFκB-p65 (Figure 4C) mRNA expression levels in the ovarian tissue of rats were analyzed. 

As shown in Figure 4 (A–C), L-1β, TNF-α, and NFκB-p65 mRNA expression levels in rats treated with 20 mg/kg TMZ alone and subjected to sham surgery did not differ statistically from the healthy group (*P*>0.05). IL-1β, TNF-α, and NFκB-p65 mRNA expression levels were significantly increased in the ischemia and I/R groups in comparison to the healthy group (*P*<0.05). These levels were significantly reduced in TMZ-administered groups (I+TMZ 10, I+TMZ 20, I/R+TMZ 10, and I/R+TMZ 20), depending on the dose as compared to the ischemia and I/R groups (*P*<0.05).

***Impacts of TMZ on histopathological changes in ovarian I/R injury***

*Light microscopy results*

**Figure 5:**
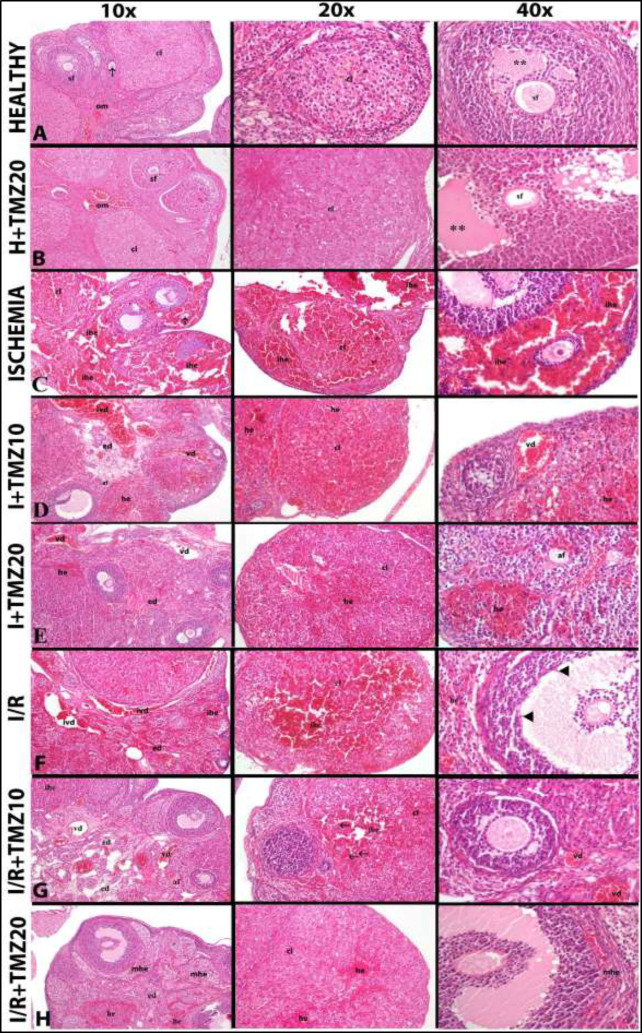
Pathologic changes: Hematoxylin-eosin staining findings of the effects of TMZ in the ovarian tissue on both ischemia and ischemia/reperfusion injury in rats

To investigate the histopathological effect of TMZ in ovarian I/R injury, ovarian tissue samples were stained using Harris’ Hematoxylin and Eosin Y staining and evaluated using a light microscope (Figure 5A-H). Also, semi-quantitative scoring of histopathologic findings was shown in Table 2.

Under light microscopy, a normal histological structure was seen in the ovarian tissues of the healthy group. The ovarian medulla one of the two basic parts of the ovary had dense blood vessels. The cortex, which is the other basic part of the ovary, contains various types of ovarian follicles and the corpus luteum. Ovarian follicles were composed of primary oocytes and antrum. Also, secondary follicles seen in different sizes and periods in the cortex were shown as sf (Figure 5-A). The ovarian tissues of rats given 20 mg/kg TMZ alone and undergoing sham surgery did not contain any pathological findings. The light microscopic findings of the histopathological appearance of this group’s cortex and medulla structures resembled those of the healthy group (Figure 5-B).

In the ovarian tissues of the ischemia group, serious pathological changes were observed. In these tissues, disintegrations, structural disorder, and cellular damage caused by ischemia were conspicuously observed. Moreover, in the cortex, dense hemorrhage areas between the follicles were remarkably seen (Figure 5-C). Examining the ovarian sections from the I+TMZ10 and I+TMZ20 groups, it was found that their structural integrity was superior to that of the ischemia group. Nevertheless, pathological alterations like edema, hemorrhage, vascular dilatations, congestion in the veins, and the result of ischemic damage persisted in the tissues of the treatment groups. When comparing the treatment groups, it was found that the I-TMZ20 group’s ovarian tissues had less edema, hemorrhage, and vascular dilatation and that TMZ20 was more effective at lessening ischemic damage (Figure 5D-E).

The I/R group’s tissue sections showed signs of severe tissue injury caused by I/R. In the cortex and medulla of this group’s ovarian tissues, intense hemorrhagic areas, intense vascular dilatation, and vein blockages were observed. Necrotic cells were found in the follicles (Figure 5-F). It was observed that the histopathological damage caused by I/R improved in TMZ-administered groups depending on the dose. In the tissue sections of TMZ-administered groups, a decrease in the intensity of vascular dilatation and hemorrhage areas was observed depending on the dose. In the I/R+TMZ10 group, general ovarian appearance was better than in the I/R group. While apoptotic cells were seen, necrotic cells were not found in ovarian tissues of the I/R+TMZ10 group (Figure 5-G). In the I/R+TMZ20 group, general ovarian appearance was better than those of both the I/R and the I/R+TMZ10 groups. While minimal hemorrhage areas were seen in the cortex, vascular dilatation and apoptotic and necrotic cells were not observed. In addition, the histopathological appearance of the I/R+TMZ20-administered group was the most similar to the healthy group (Figure 5-H).

**Table 1:** Experimental groups and design to investigate the effects of trimetazidine against ovarian ischemia/reperfusion injury in rats

Group number and name	**6 ** **hours** **before ischemia-induced**	**1 ** **hour** ** before**	**0th hour**	**2** ^nd^ ** hour**	**3** ^rd^ ** hour**	**6** ^th^ ** hour**
**1- Healthy**			Sham operation			Sacrification
**2- Healthy+TMZ 20**	TMZ	TMZ	Sham operation			Sacrification
**3- Ischemia**			Ischemia		Sacrification	
**4 -I + TMZ 10 **	TMZ	TMZ	Ischemia		Sacrification	
**5- I + TMZ 20 **	TMZ	TMZ	Ischemia		Sacrification	
**6- I/R **			Ischemia		Reperfusion	Sacrification
**7- I/R + TMZ 10 **	TMZ		Ischemia	TMZ	Reperfusion	Sacrification
**8- I/R + TMZ 20 **	TMZ		Ischemia	TMZ	Reperfusion	Sacrification

I: Ischemia; I/R: Ischemia-reperfusion; TMZ 10: 10 mg/kg Trimetazidine; TMZ 20: 20 mg/kg Trimetazidine

Levels of GSH and MDA were measured according to the modified methods with an ELISA reader. GraphPad Prism, version 5.0 was used for the statistical analysis, and the results are presented as the means ± standard deviation (SD). Comparisons between the groups were performed using one-way ANOVA and Tukey’s multiple comparison tests; *P*-value less than 0.05 was considered statistically significant. a, b, c, and d: There is no statistically significant difference between the groups in the columns symbolized with the same letter. There is a statistically significant difference between groups for columns symbolized by different letters

GSH: Glutathione; I: Ischemia; I/R: Ischemia-reperfusion; MDA: Malondialdehyde; TMZ: Trimetazidine; TMZ 10: 10 mg/kg Trimetazidine; TMZ 20: 20 mg/kg Trimetazidine

Expression of mRNAs was detected using quantitative Real-Time PCR analysis. β-actin was used as the reference gene. GraphPad Prism, version 5.0 was used for the statistical analysis, and the results are presented as the means ± standard deviation (SD). Comparisons between the groups were performed using the One-Way ANOVA and Tukey’s multiple comparison tests; P-value less than 0.05 was considered statistically significant. a, b, c, d, and e: there is no statistically significant difference between the groups in the columns symbolized with the same letter. There is a statistically significant difference between groups for columns symbolized by different letters

I: Ischemia; I/R: Ischemia-reperfusion; JAK: Janus kinase; TMZ: Trimetazidine

The expression of mRNAs was detected using quantitative Real-Time PCR analysis. β-actin was used as the reference gene. GraphPad Prism, version 5.0 was used for the statistical analysis, and the results are presented as the means ± standard deviation (SD). Comparisons between the groups were performed using the One-Way ANOVA and Tukey’s multiple comparison tests; *P*-value less than 0.05 was considered statistically significant. a, b, c, d, and e: there is no statistically significant difference between the groups in the columns symbolized with the same letter. There is a statistically significant difference between groups for columns symbolized by different letters

I: Ischemia; I/R: Ischemia-reperfusion; STAT: Signal transducer and activator of transcription; TMZ: Trimetazidine

The expression of mRNAs was detected using quantitative Real-Time PCR analysis. β-actin was used as the reference gene. GraphPad Prism, version 5.0 was used for the statistical analysis, and the results are presented as the means ± standard deviation (SD). Comparisons between the groups were performed using the One-Way ANOVA and Tukey’s multiple comparison tests; *P*-value less than 0.05 was considered statistically significant. a, b, c, d, and e: there is no statistically significant difference between the groups in the columns symbolized with the same letter. There is a statistically significant difference between groups for columns symbolized by different letters.

I: Ischemia; IL-1β: Interleukin 1 beta; I/R: Ischemia-reperfusion; NFκB-p65: Nuclear factor kappa B-p65; TMZ: Trimetazidine; TNF-α: Tumor Necrosis Factor-alpha

**Table 2:** Semi-quantitative scoring of histopathologic findings of the effects of trimetazidine against ovarian ischemia/reperfusion injury in rats

	**Healthy**	**Ischemia**	**I+TMZ10**	**I+TMZ10**	**I/R**	**I/R+TMZ10**	**I/R+TMZ20**
**Necrotic and apoptotic cells**	0-1	4	3	3	4	2-3	2
**Hemorrhage areas**	0	4	3	2-3	4	3	1-2
**Vascular dilation**	0	4	3	2-3	4	2-3	1
**Edema areas**	0	4	3	2-3	4	2-3	1

I: Ischemia; I/R: Ischemia-reperfusion; TMZ: Trimetazidine; TMZ 10: 10 mg/kg; Trimetazidine; TMZ 20: 20 mg/kg Trimetazidine, none: 0, mild: 1, medium: 2, severe:3, or very severe: 4

I: Ischemia; I/R: Ischemai-reperfusion; TMZ: Trimetazidine; TMZ 10:10 mg/kg Trimetazidine

TMZ 20: 20 mg/kg Trimetazidine. sf: secondary follicle, cl: corpus luteum, om: ovarian medulla, **: antrum, ↑: primary oocyte, ihe: intense hemorrhage, he: hemorrhage, mhe: minimal hemorrhage, pf: primary follicle, vd: vascular dilatation, vd: intense vascular dilation, ed: edema, af: atrophic follicle, arrowhead: necrotic cell, ← : apoptotic cell

## Discussion

Ovarian torsion is a gynecopathology that can impact women of all age groups, typically seen among women of reproductive age, and requires emergency surgery (42). Blood flow disturbances caused by the ovary rotating on its axis, whether with or without tubal rotation, can lead to dangerous side effects like bleeding, adhesion, thrombophlebitis, sepsis, and even death (43). Ovarian I/R injury is an extremely complex pathological problem that begins with oxygen deprivation, progresses to excessive free radical production, intensifies inflammation, and finally ends with apoptosis and cell death (44, 45). Various surgical techniques can be safe and effective in treatment following diagnosis, but research into non-surgical treatment is continuing (46). With this information in mind, the effects of TMZ in the ovarian I/R injury model in rats were evaluated biochemically, molecularly, and histopathologically.

Oxidative stress indicates a severe imbalance between free radical formation and anti-oxidant defense mechanisms, resulting in tissue damage (47). The production of ROS is viewed as a double-edged sword, implying that a specific amount of these radical and pro-oxidant chemicals is required for the proper function of some biological phenomena such as pathogen eradication and so on; however, elevated levels of ROS can cause damage to vital macromolecules such as DNA, protein, and lipids, and they have an important function in the occurrence of some pathological events such as I/R-induced ovarian injury. For instance, ROS play an important role in a variety of physiological processes, including oocyte maturation, fertilization, embryo development, and pregnancy (48-50). The primary cause of DNA damage in the ovulation process and ovarian epithelial cells is oxidative stress, which can be avoided by administering anti-oxidant agents to individuals who are at risk of developing I/R-induced ovarian damage. Several lines of evidence have suggested that oxidative stress is important in the pathophysiology of infertility. Similar research has shown that oxidative stress contributes to the development of endometriosis, as well as tubal and peritoneal infertility. Anti-oxidant mixtures can be effective in preventing ROS overproduction, and thus they can be used to treat infertility disorders caused by oxidative stress (48, 49). Infertility in rats is a known side effect of the I/R procedure. Additionally, oxidative stress has been linked to IR-related infertility [4]. Based on these findings, it is possible to speculate that anti-oxidant therapy administered to the torsioned ovary following a detorsion procedure may help to prevent ovarian dysfunction-related infertility (51). We investigated the oxidative stress factors related to ovarian I/R injury: GSH and MDA. Previous studies have shown that after ovarian I/R injury, the MDA level is increased and the GSH level is decreased (52, 53). Also, Barghi *et al*. reported that after ovarian torsion/detorsion, oxidative stress increased (54). Our research detected the levels of GSH and MDA in ovarian tissue and we found that compared with the healthy group, the MDA level was significantly increased, while GSH levels were significantly decreased in the ovarian tissue of rats in the ischemia and I/R injury groups, suggesting that after ovarian ischemia and I/R injury, oxidative stress reaction was aggravated in the ovarian tissues. Due to both severe ischemic conditions and low-dose TMZ administration, GSH levels of the ischemia group and 10 mg/kg TMZ administration ischemia group were similar. On the contrary, the noticeable rise in GSH levels and the decline in MDA levels in the ovarian tissues of both high-dose TMZ administration ischemia group and I/R injury plus TMZ groups, depending on the dose were attributed to the protective effect of TMZ, indicating the strong anti-oxidant feature. These effects of TMZ seemed to be associated with the inhibition of oxidative stress, to a lesser extent, inflammatory responses. These findings demonstrated that TMZ enhanced the oxidative situation. As a result, it reduced oxidative stress and alleviated ovarian I/R injury. Hazelhoff *et al*. reported that TMZ significantly fixed oxidative stress indicators by raising GSH levels while reducing MDA levels in a rat model of kidney injury (55). A study reported that TMZ modulated GSH and MDA levels in the testicular I/R injury model in rats (56). Researchers reported that TMZ mitigated oxidative stress parameters in a peripheral neuropathy study (57). Consistent with other findings reported in the literature, our result suggests that TMZ treatment reduces oxidant parameter generation while increasing anti-oxidant parameter generation, protecting ovarian tissue from I/R injury by controlling oxidative stress and inflammatory markers. Perhaps TMZ can be correlated with the treatment of ovarian I/R injury and ovarian I/R injury-related infertility by regulating oxidative stress.

JAK2/STAT3 is one of the important members of the JAK/STAT signaling pathway (58). The signaling cascade frequently results in a change in gene expression that can impact cellular processes like proliferation, differentiation, and homeostasis (59). JAK-STAT signaling is a crucial transducer in cardiomyocytes hypoxia injury (60), cancer (61), hematopoiesis and leukemia (62), rheumatoid arthritis (63), acute lung injury (64), central nervous system disorders (65), and obesity and diabetes (66). The key mechanisms used for such communication are JAK-STAT and TNF receptors. Deregulated JAK-STAT and TNF receptor signaling pathways can increase cytokine production and result in chronic inflammatory phenotypes (59). The activated JAK2/STAT3 signaling pathway is frequently observed in I/R conditions and is highly related to I/R-induced tissue damage (67). Changes in the JAK2/STAT3 signaling pathway affect the expression of many cytokines, such as TNF-α (68, 69). Also, the abnormal activation of the JAK2/STAT3 pathway is involved in a variety of pathophysiological processes including apoptosis (70). According to studies, the post-ischemic inflammatory response is mediated by the JAK2/STAT3 signaling pathway, which can be activated after cerebral ischemia (71, 72). Previous studies have shown that activation of the JAK2/STAT3 signaling pathway promotes the release of cytokines such as TNF-α, thereby inducing the inflammatory reaction (73). Moreover, previous studies have shown JAK2/STAT3 signaling pathway activation increase in different I/R injuries and various animal models such as cerebral I/R injury in mice (74), renal I/R injury in rats (75), and suppression of the pathway heals the tissue injury. In line with previous research, our findings indicate that the ovarian I/R injury group had higher levels of JAK2 and STAT3 mRNA expression. TMZ administration, depending on the dose, alleviated tissue injury by preventing the increase in JAK2/STAT3 signaling pathway activation caused by I/R. These findings indicate that TMZ reduced tissue injury by avoiding ovarian I/R-induced JAK2/STAT3 signaling pathway activation.

TNF-α regulates immune, inflammatory, and hematopoietic responses (76). Due to TNF-’s pleiotropic biological effects, the ovarian tissue may have undergone DNA fragmentation and apoptosis as a result of an oxidative-inflammatory response **(**77**)**. I/R-induced ROS production causes the activation of NF-kB (78). NF-κB activation increases the transcription of pro-inflammatory cytokines including tumor necrosis factor-alpha (TNF-α) (79). TNF-α can stimulate the production of other inflammatory markers, including IL-1β, and aggravate the damage of tissues and organs (80). In light of this information, in this study, the mRNA expression of IL-1β, TNF-α, and NF-κβ in ovarian tissues after ovarian I/R injury and treatment with TMZ were investigated to appraise the potential medicinal importance of TMZ in ovarian I/R injury. The present results showed that TMZ corrected changes in the anti-inflammatory parameters due to ovarian I/R injury, indicating the strong anti-inflammatory feature of TMZ. The ovarian I/R injury group had higher levels of IL-1β, TNF-α, and NF-κβ mRNA expression. These results are consistent with previous research that found ovarian I/R injury increases cytokine levels including IL-1β, TNF-α, and NF-κβ (81, 82). The increase in cytokine expressions in the ovarian I/R injury group decreased with the TMZ administration depending on the dose, in our study. These findings indicate that TMZ reduced tissue injury by avoiding ovarian I/R-induced increases in cytokine levels. As a result, the anti-inflammatory effect of TMZ appears to be related to the reduction of oxidative damage. In recent years, TMZ has been reported to be associated with several signaling pathways such as TNF-α (83, 84). In addition, TMZ ‘s anti-inflammatory effects, including suppression of pro-inflammatory cytokines, have earlier been shown in various animal models. A study demonstrated that TMZ improves neuroinflammatory cytokines such as TNF-α and NF-κB p65 in folic acid-induced acute ovarian injury in mice (85). Tanoglu *et al.* demonstrated that TMZ modulated NF-κβ and IL-1 β levels in experimental sepsis rat models (86). Researchers reported that TMZ alleviated anti-inflammatory markers including TNF-α and IL-1β parameters in the neurotoxicity model in rats (87). Consistent with the previous studies, our results suggest that TMZ reduced tissue injury by preventing the increase in IL-1β, TNF-αm, and NF-κβ levels due to I/R.

Ovarian I/R has the potential to set off inflammatory cascades that could disrupt microcirculation and harm the vascular endothelial cells that are primarily responsible for ovarian tissue damage (88). Increased ROS levels cause granulosa cell apoptosis, endothelial destruction, and DNA damage (89, 90). Additionally, prolonged I/R injuries may harm cells and result in autophagy, apoptosis, necroptosis, and necrosis (91). The pathophysiological mechanisms that cause necrosis are thought to be unregulated and uncoordinated. Apoptosis, unlike necrosis, is a regulated process with distinct underlying signal transduction mechanisms. Apoptosis is a type of cell death that uses a lot of energy (92). In light of this information, number of necrotic and apoptotic cells, size of edema, and hemorrhage areas as well as vascular dilation in ovarian tissues were evaluated. Regarding the histopathological scores in the current investigation, the histological total damage score was quite high in the ovarian I/R injury group, while the score was reduced in the 10 and 20 mg/kg of TMZ administration groups. In the I/R injury group, ovarian tissues also showed severe pathological alterations. The ovarian I/R injury group displayed hemorrhage, edema, vascular dilatation, extensive inflammatory cell infiltration, and necrotic and apoptotic changes. However, in the groups that received 10 and 20 mg/kg of TMZ, the histological aspect of the ovarian tissues was nearly normal. Our biochemical and molecular findings were corroborated by our histology findings. Our result suggests that TMZ corrected ovarian tissue injury by improving histopathologic damage due to I/R. 

TMZ may be considered a potential therapeutic agent in addition to surgery in the clinical treatment of ovarian I/R injury and related infertility. These findings may provide a mechanistic basis for using TMZ to treat I/R-induced ovarian injury.

## Conclusion

We have demonstrated that TMZ significantly reduced ovarian I/R injury and may help protect the ovaries from I/R-induced injury in humans based on the biochemical, molecular, and histopathological findings. TMZ might be interfering with anti-oxidant and anti-inflammatory processes that are important in ovarian I/R procedures. The protective effects of TMZ against ovarian I/R injury can be explained by (1) lowering immunopositivity of inflammatory cytokines, such as IL-1β, NF-κβ-p65, and TNF-α, thereby affecting the JAK/STAT signaling pathway (2) improving oxidative stress-associated variables like GSH and MDA, and (3) reducing lengthy pathological modifications related to I/R-induced ovarian injury. 

## Information

A part of this study was presented orally and online at the ISARC 4. Internatıonal Dicle Scientific Research and Innovation Congress on April 18-19, 2023. No financial support has been received for this study.

## Authors’ Contributions

TN Y, Z H, and E C designed the experiments; TN Y, Z H, E C, E T, B O, and A B performed experiments and collected data; TN Y, Z H, E C, and E T discussed the results and strategy; TN Y, Z H, and E C supervised, directed, and managed the study; TN Y, Z H, E C, E T, B O, and A B approved the final version to be published.

## Disclosure Statement

***Financial interest***

This declaration is not applicable. The authors declare that no funding, grants, or other support was received for this study.

## Conflicts of Interest

The authors declare that they have no conflicts of interest of financial or personal nature.

## Data Availability Statement

The data underlying this article are available in the article and its online supplementary material.
